# One-Year Follow-Up of Seroprevalence of SARS-CoV-2 Infection and Anxiety among Health Workers of a French Cancer Center: The PRO-SERO-COV Study

**DOI:** 10.3390/ijerph20115949

**Published:** 2023-05-25

**Authors:** Brice Richez, Coralie Cantarel, Françoise Durrieu, Isabelle Soubeyran, Julie Blanchi, Simon Pernot, Camille Chakiba Brugère, Guilhem Roubaud, Sophie Cousin, Gabriel Etienne, Anne Floquet, Florence Babre, Julie Rivalan, Caroline Lalet, Marine Narbonne, Yaniss Belaroussi, Carine Bellera, Simone Mathoulin-Pélissier

**Affiliations:** 1 Department of Anesthesia—Intensive Care, Institut Bergonié, Comprehensive Cancer Center, F-33000 Bordeaux, France; 2 Inserm CIC1401, Clinical and Epidemiological Research Unit, Institut Bergonié, Comprehensive Cancer Center, F-33000 Bordeaux, France; 3 Department of Biopathology, Institut Bergonié, Comprehensive Cancer Center, F-33000 Bordeaux, France; 4 Department of Medical Oncology, Institut Bergonié, Comprehensive Cancer Center, F-33000 Bordeaux, France; 5 Hematology Department, Institut Bergonié, Comprehensive Cancer Center, F-33000 Bordeaux, France; 6 Department PRISME, Institut Bergonié, Comprehensive Cancer Center, F-33000 Bordeaux, France; 7 Epicene Team, UMR 1219, Bordeaux Population Health Research Center, University Bordeaux, Inserm, F-33000 Bordeaux, France

**Keywords:** SARS-CoV-2, COVID-19, health workers, antibody, seroprevalence, cancer center, anxiety, vaccination hesitancy

## Abstract

Infection of SARS-CoV-2 among health workers (HWs) in contact with cancer patients has been a major issue since the beginning of the pandemic. We aimed to assess the serological immune status of SARS-CoV-2 infection among these HWs. A prospective cohort study was initiated in the comprehensive cancer center of the Nouvelle-Aquitaine region (NA, France). Volunteer HWs working on March 2020 without active infection or symptoms of COVID-19 completed a self-questionnaire and had a blood test at inclusion, at 3 and 12 months. Positive serological status of SARS-CoV-2 infection was defined by anti-nucleocapsid antibodies and/or IgG anti-spike antibodies, except at 12 months due to vaccine. Half of the HWs were included (N = 517) and 89% were followed for three months (N = 500) and one year (N = 462). Seroprevalence of SARS-CoV-2 infection was 3.5% (95% CI: 1.9–5.1), 6.2% (95% CI: 4.1–8.3), and 10% (95% CI: 7.2–12.7) on June–September 2020, September 2020–January 2021, and June–October 2021, respectively. At 12 months, 93.3% had detectable antibodies with 80% vaccinated in the first three months of vaccine availability. The COVID-19-free policy of the institution, respect for barrier gestures, high and early vaccination of HWs, and low prevalence of SARS-CoV-2 in NA may explain the low rate of seropositivity among the HWs of the Institut Bergonié.

## 1. Introduction

COVID-19 was caused by the severe acute respiratory syndrome coronavirus-2 (SARS-CoV-2) and led to a pandemic situation from the end of 2019, with more than 764 million confirmed cases and nearly 7 million deaths by April 2023 worldwide [1,2]. The health and prevention systems had to adapt and the vaccination at the beginning of 2021 helped to control the incidence of severe cases. The first two years of the pandemic were nonetheless marked by five successive waves and multiple lockdowns in Europe.

One of the concerns at the beginning of the epidemic was the proportion of asymptomatic cases. It was heterogeneous but globally estimated to be 40.5% (95% CI, 33.5–47.5%) [3]. Systematic testing was not performed in the general population in most European countries including France. However, serological tests detecting antibodies (Abs) directed against spike protein (S) and nucleocapsid (N), detectable from day 8 to 11 after contamination may have a place in epidemiological surveillance by identifying people who are or have been in contact with the virus [4,5].

The issue of immune response for the population and especially in contact with vulnerable populations has been quickly a major concern. Most studies focused on seroprevalence among health workers (HWs), especially after the first wave of the COVID-19 pandemic. A meta-analysis based on 25 studies (168 200 HWs) in Europe, Asia, and North America with blood samples from March to August 2020 reported a variation of seropositivity from 1.1% to 35.4%, inducing a pooled seroprevalence of 8% (95% CI 6–10%) with high heterogeneity (I2 = 100%, *p* < 0.001) [6].

Some studies focused on seroprevalence among health workers (HWs) in contact with cancer patients, especially after the first wave of the COVID-19 pandemic. They reported heterogeneous seroprevalence of SARS-CoV-2 from 1.8% to 18.4% between March and June 2020, as a result of variation in the performances of serological tests at the beginning of the pandemic crisis, disparities between countries in the prevalence of SARS-CoV-2 infection, and management of the COVID-19 crisis [7,8,9,10,11,12,13,14,15]. Out of them, four studies focused on health workers at French cancer centers, but the incidence rate of SARS-CoV-2 was also heterogeneous between regions [16]. In the PAPESCO-19 study, 940 health workers from four cancer centers located in the western, eastern, and center (Angers, Nantes (region Pays de la Loire), Nancy (region Grand-Est), Clermont-Ferrand (region Auvergne-Rhône-Alpes)) were included from June to November 2020 [7]. The rate of COVID-19 positivity (RT-PCR) or seroprevalence (IgG anti-N) of a previous infection of SARS-CoV-2 was 9.5%. F. Anna et al. reported in a prospective cohort study an 11% (95% confidence interval (CI): 9.7–12.6) seroprevalence (IgG anti-N and IgG anti-S) among 1847 HWs tested between April and July 2020 on three sites of the Institute Curie in Paris [12]. A short follow-up four to eight weeks later showed a decline in anti-N and anti-S IgG titers and neutralization activity in pseudo-virus assay declined by 31%, 17%, and 53% respectively among health workers who were retested. In two cross-sectional studies, Ladoire et al. assessed seroprevalence (Total: IgA, IgM, and IgG) to SARS-CoV-2 in the Georges-Francois Leclerc Cancer Centre, the comprehensive cancer center of Dijon (300 km southeast of Paris). From May to June 2020 (canSEROcov study), seroprevalence was estimated to be 1.8% among 663 health workers (medical and non-medical) [13]. On January 2021 (canSEROcov-II study), it was 15.1% (76/502) [14]. For more than 80% of health workers who participated in the two cross-sectional studies, the evolution of seroprevalence among this center was available on a 9 month follow-up.

However, these studies are cross-sectional or with short follow-ups and no data are available for the third wave of the COVID-19 pandemic (March–April 2021).

In France, from 2020 to the middle of 2021, the first three waves of the pandemic induced three lockdowns (March–May 2020, October–December 2020, April–May 2021) and the closure of public facilities and most businesses. The negative psychological impact of quarantine during recent epidemics (SARS, Ebola, H1N1) was claimed to have occurred in several populations [17]. A systematic review reported that health workers had the highest prevalence of anxiety linked to COVID-19 [18]. Some meta-analyses reported pooled prevalence of anxiety between 15.5% and 50.7% in HWs [19,20,21,22]. Few studies investigated anxiety among French HWs in contact with cancer patients. A French national survey (4–14 May 2020) reported that 32% of medical oncologists and radiation therapist residents who responded were anxious [23]. Longitudinal data are required to investigate the evolution of anxiety among health workers in cancer centers following the successive waves of the COVID-19 crisis.

We proposed the PRO-SERO-COV study, with the aim to follow the serological immune status of a previous infection of SARS-CoV-2, among health workers from a cancer center localized in the southwest of France. The prevalence and development of anxiety disorders among these professionals were also investigated in an optional study.

## 2. Materials and Methods

### 2.1. Study Design and Participants

A prospective longitudinal cohort study was conducted at the Institut Bergonié (Bordeaux), a comprehensive cancer center in Nouvelle-Aquitaine (NA, France). From June to September 2020, all HWs (medical and non-medical), 18 years or older, working in the center from at least March 2020 were invited to participate in the study, by a poster and mail campaign. HWs declaring symptoms suggestive of COVID-19 or documented infection by SARS-CoV-2 within 10 days and women declaring to be pregnant or breastfeeding were not included.

For each voluntary participant, all data were anonymized based on the participant’s inclusion number provided at the time of signing the informed consent form with the investigating physician. Thereafter, HWs were followed at inclusion, 3 and 12 months. Each visit included a blood sample taken by a nurse and two self-administered surveys in paper format, with one for the optional anxiety study. Self-questionnaires could be completed by the participant at the time of the visit or later. Participants were considered “lost to follow-up” and “survey unrecoverable” after three unsuccessful attempts at one-week intervals.

A minimum of 460 participants was needed at inclusion to estimate a proportion of 5% (low estimate) of HWs with positive serological tests (IgG or total) with a precision of estimates of 2% (Wald method, approximation by the normal law).

All data were collected and treated in accordance with Good Clinical Practices and General Data Protection Regulation. The employer had no knowledge of the HWs who agreed to participate in the study and individual data collected. This study was approved by the Ethics Committee (CPP-Sud Mediterranée/reference CNRIPH:20.05.06.62519/sponsor reference: IB 2020-01) and was registered at ClinicalTrials.gov Identifier: NCT04426006.

### 2.2. Study Questionnaires

We collected information on housing (number of people, area, location), previous SARS-CoV-2 infection (date, symptoms, diagnosis tests), contacts with symptomatic people, and lifestyle habits at work and in private life (travel and travel mode, application of barrier gestures). Demographic characteristics (age, sex), health status (overweight, smoking status, medical history), diploma, activity domain, and previous influenza vaccination were also collected at inclusion. The wish to be vaccinated against SARS-CoV-2 was asked of each participant at the inclusion and the 3 month visits. As a vaccine was available from January 2021, participants have to indicate if they have been vaccinated at the 12 month visit.

Anxiety was assessed using the Generalized Anxiety Disorders-7 (GAD-7) questionnaire, which focused on anxiety symptoms in the past 2 weeks and was validated in the French language [24,25]. This questionnaire was composed of 7 items scored on a three-point Likert scale (0 = not at all to 3 = nearly every day). The total score was categorized into the following levels of anxiety (0–4 = no, 5–9 = mild, 10–14 = moderate, 15–21 = severe). GAD is considered present for scores above 10 points.

### 2.3. Serological Assay

Blood samples were analyzed by the clinical biology unit of the center through two tests.

Total antibodies (IgG and IgM) directed against the N (nucleocapsid) protein of the virus were determined by ECLIA on a Cobas^®^ 8000 (Roche Diagnostic, France) with the Elecsys Anti-SARS-CoV-2 kit, which offers good performance (sensitivity: 100 % (95% CI: 88.1–100) and specificity: 99.81 (95% CI: 99.65–99.91)). Results were dichotomized based on a cutoff index (COI; signal sample/cutoff) as negative (COI < 1.0) or positive (COI ≥ 1.0). This test was validated by the Haute Autorité de Santé [26].

The serum was also tested using ELISA with the SARS-CoV-2 (IgG) ELISA Kit (EuroImmun, France; 87.5% to 100% sensitivity 20 days after infection and 99.3% specificity).

Briefly, the 1/101 diluted sample and positive and negative controls were distributed into 96-well plates coated with the S1 domain of the SARS-CoV-2 Spike protein and revealed with HRP-labeled anti-human IgG-specific antibody. The microplates were then assessed according to the manufacturer’s specific instructions. Absorbance at 540 nm was measured on a Multiskan FC photometer (ThermoFicher Scientific, Waltham, MA, USA).

Positive serological status indicating a previous infection of SARS-CoV-2 was defined as a positive ECLIA test and/or positive ELISA IgG test. As SARS-CoV-2 vaccines were based on the Spike Glycoprotein, only positive ECLIA tests were considered at the 12 month visit [27].

### 2.4. Statistical Analysis

At each time point, descriptive analyses were conducted on participants with a blood sample and the main questionnaire available. Qualitative variables were described as numbers and percentages. Quantitative variables were described as mean ± standard deviation or median (range). Missing data were reported.

The seroprevalence of a previous infection of the SARS-CoV-2 was reported with the associated 95% confidence interval (CI, Wald method normal approximation) at each visit. Contact with patients was a factor associated with seropositivity of SARS-CoV-2 and being a non-care health worker was a factor in anxiety levels [6,19]. Hence, we conducted an exploratory analysis comparing the seroprevalence (positive/negative) of a previous infection of the SARS-CoV-2 and anxiety level (no/mild/moderate/severe), according to activity domains (patient care/other/missing), using the two-tailed chi-squared test or Fisher exact test at the 5% significance level.

We relied on the REDcap 9.1.3 system (Vanderbilt University) and SAS 9.4 (Institute Inc., Cary, NC, USA) software for data collection and statistical analysis, respectively.

## 3. Results

### 3.1. Characteristics and Exposition to SARS-CoV-2

**Figure 1:**
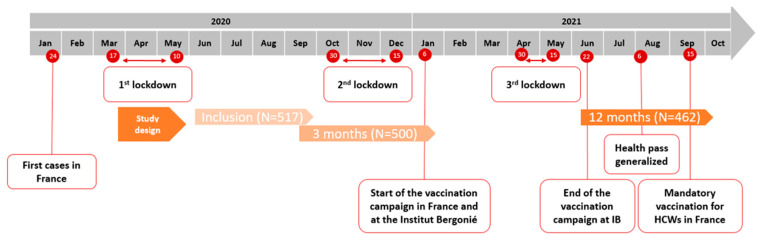
The PRO-SERO-COV study during the first three waves of the COVID-19 crisis and associated events in France.

From 18 June 2020 to 28 September 2020, out of 1085 HWs in the center, 526 (48.5%) agreed to participate in the study. Nine participants were excluded, with one not employed at the beginning of the first lockdown and eight with a questionnaire and/or blood sample not available. Hence, 517 HWs were analyzed between the first and the second lockdown, and nearly 90% were followed during one year: 500 HWs from 10 September 2020 to 26 January 2021 and 462 from 10 June 2021 to 4 October 2021 (Figure 1).

At inclusion, most of the participants were female (83.4%) and the mean age was 42 years (SD: 11) (Table 1). With a majority of subjects living in couples or alone with children, the median number of people in the household was 3 (range: 1–7). More than half of the participants were working in patient care (59.2%). Four in five participants did not report any comorbidities (n = 413, 79.9%).

### 3.2. Barrier Gestures and Vaccine Hesitancy

**Figure 2:**
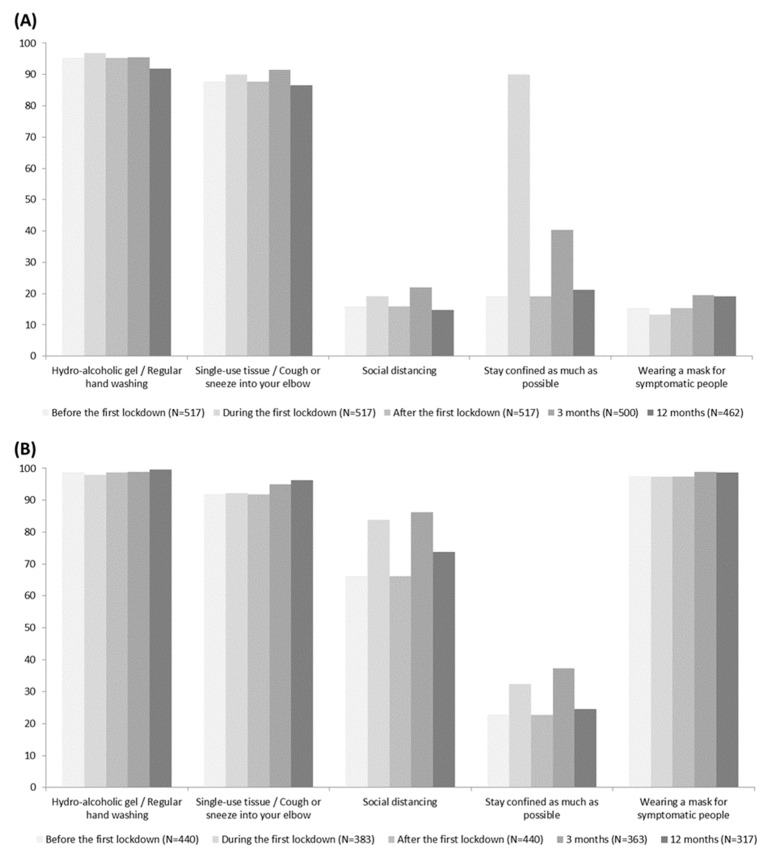
Barrier gestures during follow-up. (**A**) Barrier gestures at home (**B**) Barrier gestures at work (if full or partial work on site). Barrier gestures before the first lockdown, during the first lockdown, and after the first lockdown were collected at the inclusion visit.

Barrier gestures were assessed before, during, and after the first lockdown, at the 3 month and 12 month visits at home for all participants and at work for those working on site (383 HWs during the first lockdown, 440 after the first lockdown, 363 at the 3 month visit, 317 at the 12 month visit) (Figure 2). Regular use of hydro-alcoholic gel and/or regular hand washing were applied by more than 90% of the HWs at home (96.7% during the first lockdown, 95.2% after the first lockdown, 95.4% at 3 months, 91.8% at 12 months) and work (97.9%, 98.6%, 98.9%, 99.7%, respectively). Similarly, most of HWs declared using single-use tissues, discarding them after use, and/or coughing or sneezing into their elbow (at home: 89.9% during the first lockdown, 87.6% after the first lockdown, 91.4% at the 3 month visit, 86.6% at the 12 month visit; at work: 92.2%, 91.8%, 95%, 96.2%, respectively) and wearing a mask at work (97.4%, 97.3%, 98.9%, 98.7%). At home, less than 20% declared social distancing of at least one meter (19.0% during the first lockdown, 15.9% after the first lockdown, 22% at three months, 14.7% at 12 months) and wearing a mask for symptomatic people (13.3%,15.3%, 19.4%, 19.0%). Staying home as much as possible depended on the first and second lockdowns (89.9% during the first lockdown, 19.1% after the first lockdown, 40.4% at three months, and 21.2% at 12 months).

At work, barrier gestures were applied by 90% or more, except for gestures concerning distancing, which were fluctuating according to the period. Social distancing of at least one meter from colleagues was applied by more than one-third of the HWs during the study (83.8% during the first lockdown, 66.1% after the first lockdown, 86.2% at three months, 73.8% at 12 months). Moreover, 32.4%, 22.7%, 37.2%, and 24.6% declared staying in the office as much as possible during the first lockdown, after the first lockdown, at 3 month visit, and at 12 month visit, respectively.

Regarding vaccination, at inclusion, 232 (44.9%) HWs indicated that they would be vaccinated if a SARS-CoV-2 vaccine was developed. Further, 51 (9.9%) of HWs would refuse and 232 (44.9%) had no opinion, leading to a vaccine hesitancy rate of 54.7%. Between September 2020 and January 2021, 65.4% would refuse to be vaccinated (15.8%) or did not know (49.6%). At the 12 month visit, 429 (92.9%) HWs declared having received at least one dose; of those, 356 (82.9%) received them in January–March, 62 (14.5%) in April–June, and 9 (2.1%) in July–August. For two (0.5%) participants, this information was unknown. Out of the 33 HWs not vaccinated, 11 (33.3%) intended to be vaccinated, 8 (24.3%) did not know, and 7 (21.2%) did not intend to be vaccinated.

### 3.3. Seroprevalence against SARS-CoV-2

On June–November 2020, 18/517 HWs had a positive serological status to a previous infection of SARS-CoV-2. Out of them, five (27.8%) declared previous infection of SARS-CoV-2 with confirmation by RT-PCR for three, five (27.8%) did not know, and eight (44.4%) declared no previous infection of SARS-CoV-2. Of the seropositive paricipants who declared previous infection of SARS-CoV-2 or did not know (n = 10), 80% reported at least one symptom. Headaches, diarrhea, ageusia, anosmia, and fatigue were the main symptoms reported by 50% of them.

Between September 2020–January 2021, 31/500 participants were positive, with 18 (3.6%) incident cases. Out of the incident cases, 15 (83.3%) declared a symptomatic previous infection since the last visit, confirmed by RT-PCR for 11. Fatigue (93.3%), anosmia (86.7%), cough (73.3%), ageusia (73.3%), respiratory difficulties (66.7%), headaches (66.7%), and muscle aches and pain (66.7%) were the main symptoms.

In June–October 2021, 431/462 (93.3%, 95% CI: 91.0–95.6) HWs had a seropositive status to SARS-CoV-2. This was related to vaccination for 385/462 workers (83.3%). The remaining 46 workers were positive for anti-N antibodies with 26 (5.6%) positive for a previous infection since inclusion (n = 13) or 3 month visit (n = 13). Out of the 20 incident cases, 17 (85.0%) reported previous infection of SARS-CoV-2 since the previous visit, confirmed by RT-PCR for 12 (70.6%) and symptomatic for all except one (94.1%). Fatigue (87.5%), fever (81.3%), headaches (81.3%), respiratory difficulties (62.5%), and asthenia (62.5%) were the main symptoms.

Hence, the seroprevalence of a previous infection of SARS-CoV-2 was 3.5% (95% CI: 1.9–5.1), 6.2%, (95% CI: 4.1–8.3), and 10% (95% CI: 7.2–12.7) at inclusion, 3 month, and 12 month visits, respectively. At inclusion, 3 month, and 12 month visits, following data collected at the first visit with a positive serological status of previous infection of SARS-CoV-2, no previous infection or asymptomatic previous infection was reported for 10/18 (55.5%), 20/31 (64.5%), and 25/46 (54.3%) and no contact with any person(s) having suspected or confirmed symptoms was reported for 8/18 (44.4%), 13/31 (41.9%), and 25/46 (54.3%), respectively.

There was no difference or seroprevalence of previous infection of SARS-CoV-2 and activity domain (Table 2).

### 3.4. Anxiety

About 90% of the HWs participated in the optional study assessing anxiety (89% at inclusion, 88.4% at 3 months, and 89.4% at 12 months) (Table 3). At each time, about 10% of them reported feeling nervous, anxious, or on edge on more than half the days (10.2%, 10.0%, and 12.1%, respectively) over the last two weeks. More than 30% reported feeling nervous, anxious, or on edge (42.4%, 35.7%, and 31.2%), having trouble relaxing (36.1%, 31.4%, and 33.2%), or becoming easily annoyed or irritable (39.1%, 31.7%, and 29.5%) several days over the last 2 weeks. Anxiety profile was similar between each visit with a median score of 2 (range: 0–19 at inclusion and 12 months and 0–21 at 3 months) and 7.8%, 11.0% and 7.2% HWs had generalized anxiety disorders at inclusion, 3 month, and 12 month visits, respectively.

**Figure 3:**
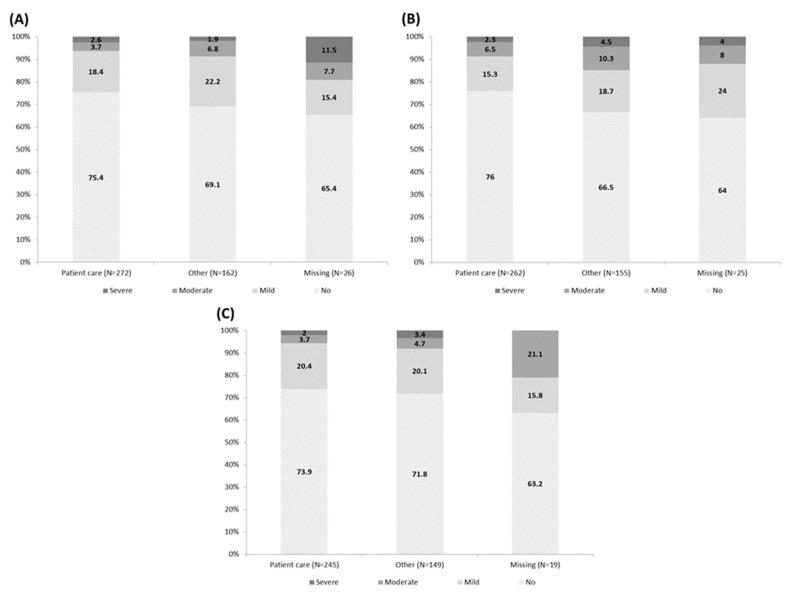
Generalized Anxiety Disorders-7 following activity domain. (**A**) Inclusion visit, (**B**) 3 month visit, (**C**) 12 month visit.

There was no difference in the prevalence of generalized anxiety disorders between HWs working in patient care and those who were not at inclusion (6.3% vs. 8.7%, *p* = 0.0545), 3 month (8.8% vs. 14.8%, *p* = 0.1544), and 12 month (5.7% vs. 8.1%, *p* = 0.0520) visits (Figure 3).

## 4. Discussion

Nearly half of the professionals at the Institut Bergonié agreed to participate in this longitudinal prospective cohort study. The seroprevalence rate of a previous infection of SARS-CoV-2 was lower or equal to 10% during the study period, which included the second and third containments. These results were similar to those observed in the general population of the Nouvelle-Aquitaine (NA) region, which had the lowest prevalence of COVID-19 infection in France during the study period [28,29].

To avoid community contact with COVID-19, the Institut Bergonié led a strengthened COVID-19-free policy. Until January 2022, patients with suspected SARS-CoV-2 infection were not treated in the center. In case of diagnosis of infection in the hospital, they were rerouted to peripheral centers (Bordeaux University hospital) or isolated for those undergoing radiotherapy (radiotherapy for COVID-19 was performed exclusively on the last time slot of the day). Other measures were implemented such as eviction of HWs with suspected SARS-CoV-2 infection, temperature taking at the entrance of the hospital for all, phone and hand disinfection at the entrance of the center, and reminders of barrier gestures (poster and email) with regular audits. Only two clusters were observed in the center during the study period involving a total of less than 30 HWs.

Most of the HWs included in the study reported having applied barrier gestures at work. However, at home, few HWs declared social distancing of at least one meter or wearing a mask for symptomatic people. Household contamination cannot be excluded as supported by other studies [30,31]. The similar seroprevalence in Nouvelle-Aquitaine in our study, the absence of association between seroprevalence and the activity domain, and the declaration of barrier gestures, could potentially be explained by COVID-19 household contamination.

Given that seroprevalence was correlated to the local infection rate, it is difficult to compare our results to other regions in France or other countries [6,7,8,9,10,11,12,13,14,15,16]. Nevertheless, the rate of asymptomatic cases was similar to that reported in the literature [3]. Out of the 18 HWs with positive serological status due to SARS-CoV-2 infections at inclusion, 12 (66.7%) were positive for IgG anti-N antibodies in at least one year, and one was lost to follow-up after baseline assessment. Most studies reported that at least 80% of HWs remained seropositive to SARS-CoV-2 for at least 6 months but it can vary by age, ethnicity, and prior symptom history [31,32,33].

Before the availability of an anti-SARS-CoV-2 vaccine, more than half of the HWs reported reluctance to vaccination, similar to the French general population [34]. At the three-month visit, on exploratory univariate analysis, factors associated with vaccine acceptancy were male (58% vs. 28%, Chi-squared test *p* < 0.001), two-year student or more (37.9% vs. 17.4% for less than two years, Fisher test *p* = 0.0001), working in patient care (38% vs. 26% for those who not, Chi-squared test *p* = 0.0249), and prior vaccination against influenza (43.2% vs. 20.0% for those who not, Chi-squared test *p* < 0.001). These factors were reported by most studies including three meta-analyses [35,36,37,38,39,40,41,42]. Older age was also reported to be associated with vaccine acceptancy but not in our study [37,39,41]. In addition, trust in governments and health authorities, access to reputable information sources about COVID-19 and COVID-19 vaccines, fear of COVID-19, and perceived individual risk were other factors associated with vaccine acceptance, but not investigated in our study [39,41,43].

Despite the rate of vaccine hesitance, in the first three months of vaccine availability, more than 80% received their first dose, compared to 13.9% in the general French population, and 93% of the participants declared having received at least one dose of the anti-SARS-CoV-2 vaccine at the 12 month visit [44]. This suggests a strong involvement of HWs in preventive measures when available and before the announcement of mandatory vaccination of HWs in France on July 2021.

Supported by a high response rate, a low and stable level of anxiety was observed during the study, with less than 12% of the HWs having generalized anxiety. Some meta-analyses reported pooled anxiety levels (30% to 36%) but interpretation is limited due to the high heterogeneity between studies (8% to 100%) [18,20]. Women, nurses in contact with COVID-19 patients, outbreak, occupational and personal stress, and fewer years of post-training work experience were associated with a higher level of anxiety [45,46,47]. As no data before the outbreak were available, the association between the COVID-19 crisis and the level of anxiety could not be explored in our population. The absence of association between the prevalence of generalized anxiety disorders and working in patient care may be hypothesized by the low prevalence of COVID-19 in the NA region, and the strengthened COVID-19-free policy at Institut Bergonié may have contributed to maintaining a low level of anxiety among health workers during the study period.

Our study has several strengths. To our knowledge, this is the first longitudinal cohort study evaluating seroprevalence of previous infection of SARS-CoV-2, vaccine hesitance, and anxiety during the three first waves of the COVID-19 outbreak in a French cancer center. We observed low attrition (10.6%) and based on human resource department data, the study population was comparable in terms of activity domain and sex, with a few more women (83% in the study vs. 73% in the cancer center), supporting internal validity. However, the generalization of our results to HWs working in French cancer centers is limited by the monocentric assessment, the variation in the prevalence of the outbreak between regions, and the variability in its management between centers.

This study also has some limitations. Self-declaration of barrier gestures and previous COVID-19 infections represented a major limitation, in particular, due to recall bias, but it did not impact estimations of seroprevalence of a previous infection of SARS-CoV-2. Indeed, it was assessed blinded to the questionnaire; centrally; and using automated, reproducible, sensitive, and specific tests.

Underestimation of the seroprevalence of SARS-CoV-2 infection cannot be excluded due to possible infection and seronegativation before inclusion or between visits. Nevertheless, the study began only two months after the peak of the first wave and each visit corresponded to an event of the COVID-19 outbreak.

The GAD-7 questionnaire was validated in the general population in a context outside of an exceptional situation such as a pandemic. Since the GAD-7 focuses on feedback over the past two weeks, we cannot guarantee the performance of this questionnaire in periods affected by multiple changes over short periods. The rate of anxiety may have been underestimated among responders.

## 5. Conclusions

Seroprevalence of a previous SARS-CoV-2 infection remained low during the first three waves of the COVID-19 crisis, among HWs working or not in care, in the Institut Bergonié. In addition to the strengthened COVID-19-free policy of the cancer center, a great involvement of HWs in preventive measures was observed. This study provides additional longitudinal data to contribute to cross-regional and cross-national learning to optimize pandemic preparedness and response. Moreover, the adherence of health professionals to a study assessing anxiety in this context must be underlined and encouraged to propose additional studies in this population.

## Figures and Tables

**Table 1:** Characteristics of health workers at inclusion (N = 517).

	Eligible Population N = 517
**Age (years)**—mean (SD)	42 (11)
**Gender**	
Male	86 (16.6%)
Female	431 (83.4%)
**Last degree obtained**	
Less than two-year degree	114 (22.1%)
Two-year degree or more	392 (75.8%)
Not available	11 (2.1%)
**Activity**	
Care	306 (59.2%)
Social, educational, psychological, and cultural	10 (1.9%)
Clinical or fundamental research	48 (9.3%)
Engineering and technical maintenance	2 (0.4%)
Purchasing-Logistics	7 (1.4%)
Quality, hygiene, safety, environment	12 (2.3%)
Information systems	8 (1.5%)
Information management	71 (13.7%)
Management, management, and decision support	23 (4.4%)
Not available	30 (5.8%)
**Home life**	
Alone	96 (18.6%)
Alone with one or more children	53 (10.3%)
In couple with or without children	353 (68.3%)
With friends, roommates, family	14 (2.7%)
Not available	1 (0.2%)
**Overweight**	
Yes	88 (17.8%)
No	411 (79.5%)
Don’t know or not available	18 (3.4%)
**Smoking**	
Never smoked	259 (50.1%)
Former smoker	146 (28.2%)
Smoke	108 (20.9%)
Not available	4 (0.8%)
**Vaccinated against influenza**	
No	220 (42.6%)
Yes, 2020 and/or 2019	196 (37.9%)
Yes, 2015–2018	28 (5.4%)
Yes before 2015	39 (7.5%)
Yes, last dose unknown	29 (5.6%)
Not available	5 (1.0%)
**Heart disease**	24 (4.6%)
**Chronic respiratory pathology**	21 (4.1%)
**Dysthyroidia**	16 (3.1%)
**Immunosuppression or inflammatory-related pathology**	12 (2.3%)
**Diabetes**	6 (1.2%)
**Chronic renal pathology**	3 (0.6%)
**Cancer**	3 (0.6%)
**Other pathology**	29 (5.6%)

**Table 2:** Seroprevalence of a previous infection of SARS-CoV-2 according to activity domain.

	Patient Care		Other		Missing		*p* *
	n/N	% (95% CI)	n/N	% (95% CI)	n/N	% (95% CI)	
Inclusion	9/306	2.9 (1.0–4.8)	9/181	5.0 (1.8–8.1)	0/30	-	0.3521
3 months	19/295	6.4 (3.6–9.2)	12/176	6.8 (3.1–10.5)	0/29	-	0.4340
12 months	29/270	10.7 (7.0–14.4)	17/167	10.2 (5.6–14.8)	0/25	-	0.2626

* Fisher test 95% CI: 95% confidence interval.

**Table 3:** Anxiety among health workers—Generalized Anxiety Disorders-7 questionnaire.

	Inclusion (N = 460) n (%)	3-Months (N = 442) n (%)	12-Months (N = 413) n (%)
**Feeling nervous, anxious, or on edge**			
Not at all	186 (40.4)	212 (48.0)	216 (52.3)
Several days	195 (42.4)	158 (35.7)	129 (31.2)
More than half the days	47 (10.2)	44 (10.0)	50 (12.1)
Nearly every day	29 (6.3)	27 (6.1)	17 (4.1)
Not available	3 (0.7)	1 (0.2)	1 (0.2)
**Not being able to stop or control worrying**			
Not at all	346 (75.2)	336 (76.0)	321 (77.7)
Several days	88 (19.1)	76 (17.2)	66 (16.0)
More than half the days	19 (4.1)	16 (3.6)	20 (4.8)
Nearly every day	3 (0.7)	9 (2.0)	3 (0.7)
Not available	4 (0.9)	5 (1.1)	3 (0.7)
**Worrying too much about different things**			
Not at all	357 (77.6)	336 (76.0)	324 (78.5)
Several days	73 (15.9)	68 (15.4)	67 (16.2)
More than half the days	19 (4.1)	23 (5.2)	12 (2.9)
Nearly every day	7 (1.5)	10 (2.3)	6 (1.5)
Not available	4 (0.9)	5(1.1)	4 (1.0)
**Trouble relaxing**			
Not at all	231 (50.2)	235 (53.2)	227 (55.0)
Several days	166 (36.1)	1 (31.4)	137 (33.2)
More than half the days	42 (9.1)	40 (9.0)	31 (7.5)
Nearly every day	16 (3.5)	23 (5.2)	16 (3.9)
Not available	5 (1.1)	5 (1.1)	2 (0.5)
**Being so restless that it’s hard to sit still**			
Not at all	367 (79.8)	357(80.8)	330 (79.9)
Several days	74 (16.1)	52 (11.8)	60 (14.5)
More than half the days	13 (2.8)	20 (4.5)	15 (3.6)
Nearly every day	3 (0.7)	7 (1.6)	3 (0.7)
Not available	3 (0.7)	6 (1.4)	5 (1.2)
**Becoming easily annoyed or irritable**			
Not available	2 (0.4)	4 (0.9)	4 (1.0)
Not at all	234 (50.9)	240 (54.3)	2 (57.9)
Several days	180 (39.1)	140 (31.7)	122 (29.5)
More than half the days	28 (6.1)	40 (9.0)	38 (9.2)
Nearly every day	16 (3.5)	18 (4.1)	10 (2.4)
**Feeling afraid as if something awful might happen**			
Not at all	361 (78.5)	339 (76.7)	334 (80.9)
Several days	76 (16.5)	69 (15.6)	54 (13.1)
More than half the days	12 (2.6)	19 (4.3)	15 (3.6)
Nearly every day	9 (2.0)	10 (2.3)	7 (1.7)
Not available	2 (0.4)	5 (1.1)	3 (0.7)
**Anxiety disoder**			
No	334 (72.6)	318 (71.9)	300 (72.6)
Mild	90 (19.6)	75 (17.0)	83 (20.1)
Moderate	23 (5.0)	35 (7.9)	20 (4.8)
Severe	13 (2.8)	14 (3.2)	10 (2.4)

## Data Availability

Data are unavailable due to privacy.
